# Extending the residence period of allogeneic iPSC-derived invariant natural killer T cells in humanized mice by editing HLA class I expression

**DOI:** 10.3389/fcell.2026.1869698

**Published:** 2026-07-06

**Authors:** Yun-Hsuan Chang, Takahiro Aoki, Momoko Okoshi, Munechika Yamaguchi, Hiroko Okura, Satoko Sasaki, Yoshie Sasako, Sachiko Kira, Nayuta Yakushiji-Kaminatsui, Masashi Matsuda, Manabu Nakayama, Shinichiro Motohashi, Haruhiko Koseki

**Affiliations:** 1 Laboratory for Developmental Genetics, RIKEN Center for Integrative Medical Sciences, Kanagawa, Japan; 2 Department of Medical Immunology, Graduate School of Medicine, Chiba University, Chiba, Japan; 3 Laboratory of Medical Omics Research, Department of Frontier Research and Development, Kazusa DNA Research Institute, Chiba, Japan

**Keywords:** allogeneic rejection, HLA-C, HLA-E, invariant NKT cells, iPS cells

## Abstract

Induced pluripotent stem cell (iPSC)-derived immune cells show promise for allogeneic immunotherapies. However, their clinical efficacy can be limited by early rejection of grafted iPSC-derived cells by host T cells and natural killer (NK) cells due to HLA mismatching. In theory, disrupting HLA class I expression can prevent T cell-mediated rejection, but, in its place, NK cells can eliminate “missing-self” targets due to the absence of HLA-C and HLA-E. Therefore, we devised a strategy to extend the residence time of iPSC-derived invariant natural killer T (iPSC-derived iNKT) cells in humanized mice harboring allogeneic peripheral blood mononuclear cells (PBMCs) by genetically modifying HLA class I expression. To this end, we disrupted HLA class I expression by deleting the *β2-microglobulin* (*B2M*) gene, and then recapitulated HLA-C and/or HLA-E expression in the iPSC-derived iNKT cells. Parental and modified iPSC-derived iNKT cells were assessed for their susceptibility to T- or NK cell-mediated cytotoxicity *in vitro* and for their residence time in allogeneic humanized mice. *In vitro*, dual co-expression of HLA-C and HLA-E facilitated escape from NK cell-mediated cytotoxicity, whereas single expression of either HLA-C or HLA-E provided inconsistent protection across NK cell donors. Since allogeneic HLA-C alleles can be recognized by T cells, we examined the impact of HLA-C mismatching on T cell-mediated cytotoxicity. T cell-mediated cytotoxicity against B2M-deficient iPSC-derived iNKT cells was restored when mismatched HLA-C was re-expressed. *In vivo*, B2M deficiency extended the residence time of iPSC-derived iNKT cells in allogeneic humanized mice. This effect was further enhanced by expression of HLA-C alleles matched to donor PBMCs, whereas expression of incompatible HLA-C reduced residence time. Taking into account the limitations of the humanized mouse model in recapitulating human NK cell function, these observations highlight the dominant role of T cell-mediated mechanisms in the rejection of allogeneic iPSC-derived iNKT cells *in vivo*. This study presents a promising approach for generating iPSC-derived iNKT cells tailored for a limited master cell bank, with the potential to develop universal off-the-shelf immunotherapy.

## Introduction

Adoptive immunotherapy has emerged as a leading approach for cancer treatment, with chimeric antigen receptor (CAR) T-cell therapy being one of the most impactful applications (Patel et al., 2025). It has demonstrated remarkable success in hematologic malignancies and is increasingly being explored for solid tumors. Although autologous CAR T cell transplantation is ideal, the high manufacturing costs and limited availability of suitable cell resources restrict its broader clinical application. Thus, strategies to develop scalable off-the-shelf cell sources for allogeneic immune cell therapy are currently being explored (Deuse and Schrepfer, 2025).

Human induced pluripotent stem cells (iPSCs) have emerged as a major allogeneic source for generating various differentiated cell types for therapeutic applications (Cerneckis et al., 2024). Through reprogramming of somatic cells with defined transcription factors, iPSCs gain self-renewal capacity and retain pluripotency, thereby providing a stable and scalable platform for allogeneic transplantation. Several studies have demonstrated the ability of iPSCs to differentiate into various hematopoietic lineages, including T cells, natural killer (NK) cells, and natural killer T (NKT) cells (Iriguchi et al., 2021; Iinuma et al., 2025; Sun et al., 2025). In line with these advances, we previously established a platform to generate invariant NKT cells from human iPSCs (iPSC-derived iNKT cells), which has already progressed to clinical evaluation for their antitumor potential (Yamada et al., 2016; Aoki et al., 2023; Iinuma et al., 2025). However, immune rejection triggered by human leukocyte antigen (HLA) mismatching between donor-derived cells and recipients remains a major barrier to the clinical application of allogeneic iPSC-derived immune cell therapies.

From an immunological perspective, immune rejection is mediated through distinct pathways involving T cells and NK cells. T cells recognize mismatched HLA molecules, leading to classical alloreactive cytotoxicity. In contrast, NK cells are activated by the absence or reduction of HLA class I expression, a process known as “missing-self” recognition. NK cell activity is regulated by inhibitory receptors, including killer cell immunoglobulin-like receptors (KIRs) that interact with HLA-C, as well as CD94/NKG2A that recognizes HLA-E. Taken together, these mechanisms reveal the central challenge in HLA engineering–balancing the avoidance of T cell-mediated alloreactivity with evasion of NK cell-mediated missing-self responses.

To mitigate the risk of rejection caused by HLA mismatches, the generation of hypoimmunogenic iPSCs through disruption of HLA expression has been proposed as a strategy to reduce host immune responses and extend graft survival (Deuse et al., 2019; Han et al., 2019). In particular, knockout of the β2-microglobulin (*B2M*) gene abolishes the expression of the B2M protein, which forms a heterodimer with HLA class I molecules and is indispensable for their surface expression, is suggested as an effective approach to prevent allorecognition of iPSCs by T cells (Wang et al., 2021).

On the other hand, complete elimination of HLA class I expression renders cells susceptible to NK cell-mediated cytotoxicity. To address this issue, previous studies have suggested retaining HLA-C or re-expressing HLA-E to interact with inhibitory NK cell receptors and suppress activation through missing-self recognition (Gornalusse et al., 2017; Xu et al., 2019). However, retention of endogenous HLA-C limits broad clinical applicability, as each retained line is compatible only with recipients carrying matching HLA-C alleles. As a result, allelic diversity necessitates establishing multiple retained-HLA-C iPSC lines to achieve wide-ranging population coverage. To overcome this constraint, we pursued a strategy of re-expressing HLA-C and/or HLA-E, enabling the generation of customizable HLA-C alleles from a single parental iPSC line. Notably, however, HLA-C re-expression may reintroduce an allogeneic target for CD8 T cells (Bettens et al., 2016), leaving unresolved whether re-expression of HLA-C and/or HLA-E can improve overall persistence of iPSC-derived iNKT cells by reducing NK-cell attack without restoring prohibitive T-cell alloreactivity. Furthermore, the effectiveness of similar strategies in established iPSC-derived iNKT cells remains unclear.

In this study, we aimed to evaluate whether modulating HLA expression can enhance the resistance of iPSC-derived iNKT cells to alloreactive T and NK cell responses. Our goal was to optimize their residence time following allogeneic transplantation and to provide new insights into the development of universally hypoimmunogenic iPSC-derived immune cell sources.

## Methods

### Human specimens and isolation

All experiments were approved by the Institutional Review Board for Human Research at RIKEN under approval number 2023-5. Venous blood was collected with informed consent from donors at RIKEN. All procedures were performed in accordance with the Declaration of Helsinki and the Ethical Guidelines for Medical and Biological Research Involving Human Subjects in Japan. Donors were not selected based on cytomegalovirus (CMV) serostatus, and CMV serology was not assessed as part of donor inclusion criteria. PBMC were isolated using Ficoll-Paque medium (Cytiva, Marlborough, MA, United States), and density gradient centrifugation was performed in accordance with the manufacturer’s instructions. Briefly, diluted blood samples were centrifuged at 1,000 *g* for 10 min at 25 °C; subsequently, the mononuclear cell layer was collected and suspended in complete medium.

Human NK cells were isolated from donor PBMC using the NK Cell Isolation Kit (Miltenyi Biotec, Rhineland, Germany), according to the manufacturer’s instructions. Briefly, PBMC were resuspended in 40 μL of buffer per 1 × 10^7^ total cells, followed by addition of 10 μL of NK Cell Biotin-Antibody Cocktail per 1 × 10^7^ cells and then incubated for 5 min at 4 °C. Subsequently, 30 μL of buffer and 20 μL of NK cell MicroBead Cocktail per 1 × 10^7^ cells were added, and samples were incubated for 10 min at 4 °C. NK cells were then isolated by magnetic separation using separation columns according to the manufacturer’s protocol. The purity of isolated NK cells was assessed by staining with V450-conjugated anti-CD3 (BD Biosciences, San Jose, CA, United States) and PE-Cy7-conjugated anti-CD56 antibodies (BD Biosciences) and analysis by flow cytometry (Supplementary Figure 1A). The isolated cell population contained approximately 92% CD3^−^CD56^+^ NK cells.

To generate alloreactive CD8^+^ T cells, donor PBMCs were repeatedly stimulated with parental iPSC-derived iNKT cells carrying HLA-C*01:02/14:03. Co-cultures were maintained in complete RPMI 1640 medium (Thermo Fisher Scientific, Waltham, MA, United States) supplemented with 100U/mL hIL-2 (Shionogi, Osaka, Japan) for at least 14 days, and stimulation was repeated at least twice. CD8^+^ T cells were then enriched using the CD8^+^ T Cell Isolation Kit, human (Miltenyi Biotec). Purity of the isolated cells was analyzed by flow cytometry using V450-conjugated anti-human CD3 (BD Biosciences) and FITC-conjugated anti-human CD8 antibodies (BioLegend) (Supplementary Figure 1B).

HLA genotyping of donors was performed at the HLA Laboratory (Kyoto, Japan).

### Human iPSCs culture and genomic editing

We used iNKT-iPS cells generated previously in our laboratory (Yamada et al., 2016). iPS cells were maintained on iMatrix-511 (Matrixome, Osaka, Japan) coated plates in StemFit AK02N media (Ajinomoto, Tokyo, Japan).

To generate HLA-C or HLA-E single knock-in iPS cell clones, each transgene was targeted to the AAVS1 locus using CRISPR-Cas9-mediated integration (Oceguera-Yanez et al., 2016). For electroporation, 1 × 10^5^ iPSCs were dissociated into single cells and resuspended in 10 μL of Buffer R containing 1 μg of donor plasmid and an RNP complex composed of 0.3 μL of 50 μM crRNA:tracrRNA duplex and 0.5 μL of 62 μM Alt-R S. p. HiFi Cas9 Nuclease V3 (Integrated DNA Technologies, Coralville, IA, United States). Electroporation was performed using the Neon Transfection System at 1400 V, 20 ms pulse width, and two pulses. After electroporation, cells were plated on an iMatrix-511-coated 6 cm dish in StemFit AK02N medium. One week later, gene-edited iPS cells were sorted by FACS ARIA (BD Biosciences) using PE-conjugated anti-HLA-C (BD Biosciences) or APC-conjugated anti-HLA-E (BioLegend) antibodies, and 500 sorted cells were seeded on a 6 cm dish. Single-cell-derived colonies were picked after 7 days. To validate successful transfection, genomic PCR and flow cytometry were performed. The sequence of the AAVS1 crRNA was =GGG​GCC​ACU​AGG​GAC​AGG​AU. The donor plasmid was constructed by replacing the EGFP-luciferase gene with the B2M-HLA-C or B2M-HLA-E gene in pAAVS1-P-CAG-GFPluc2 (Addgene, Watertown, MA, United States, #80493) (Oceguera-Yanez et al., 2016) as the backbone vector. The B2M-HLA-C and B2M-HLA-E genes were synthesized using GenScript (Piscataway, NJ, United States) according to previous studies (Pascolo et al., 1997; Gornalusse et al., 2017) with reference to HLA-C *01:02:01:01 and HLA-E *01:03:01:01 in the IPD-IMGT/HLA database (https://www.ebi.ac.uk/ipd/imgt/hla/).

Dual B2M-linker-HLA-C and B2M-linker-HLA-E are fusion proteins. Integrated B2M-KO Akaluc-expressing iPS cell clones were generated by co-electroporation with piggyBac transposase and each transgene plasmid, as previously reported (Tsukiyama and Ohinata, 2014). For electroporation, 1 × 10^5^ iPSCs were dissociated into single cells and resuspended in 10 μL of R buffer containing 0.9 μg of donor vector and 0.1 μg of pCAG-hyPBase vector. Electroporation was performed using the Neon Transfection System at 1400 V, 20 ms pulse width, and two pulses. After electroporation, cells were plated on iMatrix-511-coated 6 cm dish in StemFit AK02N medium. One week later, gene-edited iPS cells were sorted by FACS ARIA using PE-conjugated anti-HLA-C (BD Biosciences) and APC-conjugated anti-HLA-E (BioLegend) antibodies, and 500 sorted cells were seeded on a 6 cm dish. To validate successful transfection, genomic PCR and flow cytometry were performed. The pCAG-hyPBase vector was provided by Dr. Ohinata from Chiba University. The donor plasmid was constructed by replacing rtTA with B2M-HLA-C or B2M-HLA-E in the backbone vector PB-(CAG-rtTA_Adv; EOS-C (3+)-EGFP-IRES-puro). Akaluc was knocked in at the AAVS1 site by the CRISPR-Cas9 system as described above. The pcDNA3 Venus-Akaluc (Iwano et al., 2018) was provided by the RIKEN BRC through the National BioResource Project of the MEXT, Japan (#RDB15781).

For each genome-editing experiment, eight candidate iPSC clones were screened. Clones were evaluated based on successful transgene integration, surface expression of HLA-C and/or HLA-E, and differentiation efficiency toward iPSC-derived iNKT cells. The clone showing the highest differentiation efficiency was selected for subsequent experiments.

### Flow cytometry

Flow cytometry analysis was performed on a FACSCanto™ II (BD Biosciences), and data were analyzed using FlowJo software (Tree Star, Ashland, OR, United States). A total of 5 × 10^4^ cells were collected, washed with 2 mL of wash buffer (D-PBS containing 2% FBS), and resuspended in 100 μL of wash buffer. Cells were then stained with 2 μL of APC-conjugated anti-human B2M (BioLegend, San Diego, CA, United States), 2 μL of BV421-conjugated anti-human HLA-C (BD Biosciences), and 2 μL of APC-conjugated anti-human HLA-E (BioLegend) antibodies for 15 min at 4 °C. Unstained cells and isotype-matched control antibodies were used to establish negative gating and verify the background staining patterns. To confirm the differentiation of iPSC-derived iNKT cells, 5 × 10^4^ cells were collected, washed, and resuspended in 100 μL of wash buffer, followed by staining with 2 μL of PE-conjugated anti-human Vα24 (Beckman Coulter, Brea, CA, United States), 2 μL of FITC-conjugated anti-human Vβ11 (Beckman Coulter), 2 μL of V450-conjugated anti-human CD3 (BD Biosciences), and 2 μL of APC-conjugated anti-human CD45 antibodies (BD Biosciences). Three μL of 7-AAD (BD Biosciences) was added to 100 μL of the resuspended cells. After lymphocytes were gated based on FSC-A and SSC-A, 7-AAD^-^ viable cells were selected, followed by CD45^+^ gating, and expression of CD3, Vα24, and Vβ11 was subsequently evaluated.

### Genomic PCR analysis

Genomic PCR was performed to confirm the integration of the single-chain HLA constructs (scHLA-C and/or scHLA-E) in B2M-KO iPS cells. Genomic DNA was isolated from WT and genome-edited iPS cells using the Wizard® SV Genomic DNA Purification System (Promega, Madison, WI, United States) according to the manufacturer’s instructions. 30 ng of genomic DNA were used as template for PCR amplification using specific primers and the KOD FX Neo kit (Toyobo, Osaka, Japan). For detection of scHLA-C integration, the forward primer 5′-TTT​ACT​CAC​GTC​ATC​CAG​CAG-3′ and reverse primer 5′-TGC​CTG​GCG​CTT​GTA​CTT​CTG-3′ were used, generating an expected product of 530 bp. For detection of scHLA-E integration, the same forward primer 5′-TTT​ACT​CAC​GTC​ATC​CAG​CAG-3′ and a reverse primer 5′-CGC​CTC​AGA​GGC​ATC​ATT​TG-3′ were used, amplifying a 791 bp fragment. PCR was performed with an initial denaturation at 98 °C for 2 min, followed by 35 cycles of 98 °C for 10 s, 63 °C for 15 s, and 68 °C for 15 s, with a final extension at 68 °C for 5 min. PCR products were resolved by 1.5% of agarose gel electrophoresis, prepared using Agarose D (NIPPON GENE, Tokyo, Japan) in 1x TAE buffer (NIPPON GENE), and visualized by staining with GelRed (Biotium, Inc., Fremont, CA, United States) (10 μL per 100 mL agarose gel). The appearance of bands of the expected size confirmed the integration of the scHLA-C and/or scHLA-E constructs.

### Differentiation of iPSCs into iNKT cells

iPSC-derived iNKT cells were generated using the OP9-DLL1 stromal co-culture system as previously described (Yamada et al., 2016; Aoki et al., 2026). iNKT-iPSC colonies were mechanically divided and then seeded on mitomycin-C (Sigma-Aldrich, St. Louis, MO)-treated overconfluent OP9-DLL1 cells in 10 cm dishes filled with 10 mL of OP9-DLL1 medium [MEMα (Life Technologies) with 20% FBS (fetal bovine serum, Gibco, Waltham, MA, United States) supplemented with 10 μM Y-27632 (FUJIFILM Wako Pure Chemical, Osaka, Japan)]. The next day and on day 7, the medium was replaced with 10 mL of fresh medium. On day 13, progenitor cells were harvested. To remove stromal cells and aggregated cells, cells were passed through an EASYstrainer (100 μm, Greiner Bio-One, Frickenhausen, Germany). Then, the cells were plated on a mitomycin-C-treated semi-confluent OP9-DLL1 cells in a dish with OP9-DLL1 medium containing 5 ng/mL of hIL-7 (R&D Systems, Minneapolis, MN, United States), 5 ng/mL of hFlt-3L (R&D), and 10 ng/mL of hSCF (R&D). On days 16 and 23, semi-adherent cells were collected and passaged into a new dish layered with mitomycin C-treated OP9-DLL1 cells. After differentiation into immature iPSC-derived iNKT cells until day 30, the cells were cryopreserved and stored in liquid nitrogen. After thawing, 2.5 × 10^5^/mL iPSC-derived iNKT cells were cultured for 9–14 days in MEMα medium (Life Technologies) supplemented with 20% human male AB serum (Access Biologicals, Vista, CA, United States), 5 ng/mL human IL-7 (Peprotech, Cranbury, NJ, United States), and 10 ng/mL human IL-15 (Peprotech) using a FlexiRoll Cell Roller (5 rpm, Argos Technologies, Vernon Hills, IL, United States).

### *In vitro* cytotoxicity assay

The K562 NK target cell line was obtained from the JCRB Cell Bank (Osaka, Japan) and maintained in RPMI 1640 medium (Thermo Fisher Scientific) supplemented with 10% FBS (Gibco), 1% HEPES (1M) (Gibco), 0.5% StemSureR 10 mmol/L 2-Mercaptoethanol Solution (×100) (FUJIFILM Wako Pure Chemical), and 1% Penicillin-Streptomycin Solution (×100) (FUJIFILM Wako Pure Chemical). Target cells, including K562 and iPSC-derived iNKT cells, were labeled with CellTrace Violet (CTV; Thermo Fisher Scientific) (1 μL CTV per 1 mL D-PBS per 5 × 10^6^ cells) according to the manufacturer’s instructions.

CTV-labeled target cells (4 × 10^4^ cells per well) were co-cultured with effector NK cells isolated from donor PBMC at an effector-to-target (E:T) ratio of 2:1 for 24 h in 100 μL complete medium per well in 96-well plates at 37 °C. Two independent experiments were performed.

Cytotoxic activity was examined by flow cytometry following staining with APC-conjugated Annexin V (BioLegend) and 7-AAD (BD Biosciences), as previously described (Aoki et al., 2020). Lymphocytes were gated based on FSC-A and SSC-A. NK cell-mediated cytotoxicity was assessed by first gating on CTV^+^ target cells, and cytotoxicity was determined by quantifying the loss of viable CTV^+^ target cells (defined as Annexin V^−^7-AAD^-^).

### *In vivo* experiments

Female human IL-7-and IL-15-expressing NSG mice (Matsuda et al., 2019) aged 6–10 weeks, maintained under specific pathogen-free (SPF) conditions at the RIKEN animal facility, were used. Mice were randomly assigned to each experimental group where feasible. 3 × 10^6^ human PBMC from healthy donors and 3 × 10^6^ Akaluc-expressing wild-type, B2M-KO, or HLA-C/E dual-expressing iPSC-derived iNKT cells were collected and resuspended in 100 µL D-PBS for intravenous administration via the tail vein per mouse. The number of mice in each group was four or five. The residence time of the transplanted cells was monitored on days 6, 12, 20, and 27 post-injection using an IVIS Spectrum system, 5 minutes after intraperitoneal administration of 200 µL of 5 mM AkaLumine-HCl (Fujifilm, Tokyo, Japan). Bioluminescent signals were automatically acquired and quantified as the total photon flux (photons/s) using Living Image software, and the values were normalized to the background. Background correction was performed using background signals measured during an overnight period when no animals were imaged. All *in vivo* experiments were approved by the Institutional Animal Care and Use Committee of RIKEN under protocol number AEY 2023-19 and were performed in accordance with institutional and national guidelines for animal care and use.

### Statistical analysis

Data were analyzed using an unpaired two-tailed Student’s t-test with equal variance assumed. A *p*-value <0.05 was considered statistically significant.

## Results

### Impact of single HLA molecule recapitulation on preventing NK-mediated cytotoxicity

**FIGURE 1:**
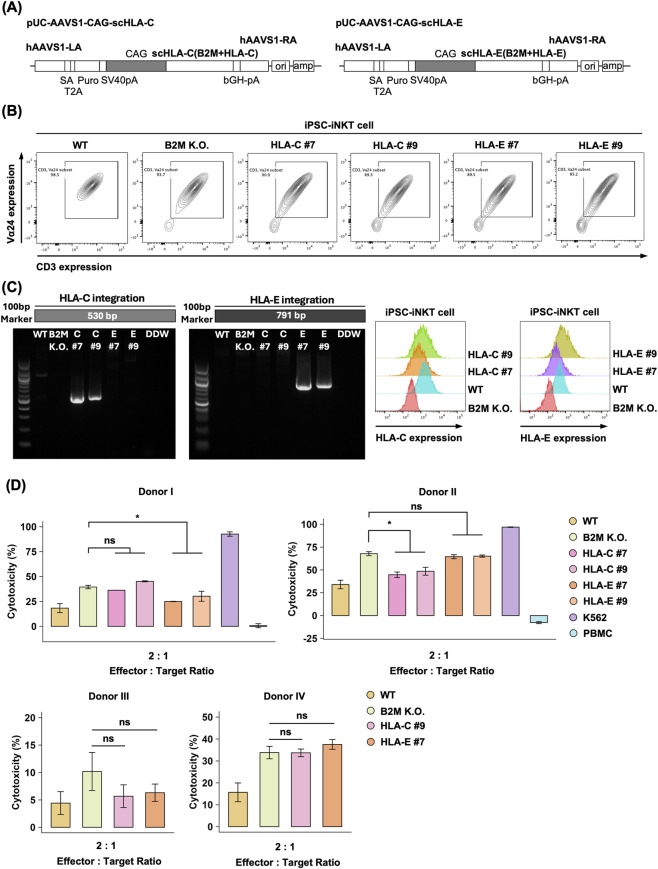
HLA-C or HLA-E single HLA molecule recapitulation is insufficient to evade NK-cell-mediated cytotoxicity. **(A)** Schematic of the AAV vectors used to edit HLA-C or HLA-E re-expressed iPSC-derived iNKT cells. **(B)** The expression of iPS-iNKT cell markers CD3 and Vα24 on the WT, B2M-KO, and HLA-C or HLA-E re-expressed iPSC-derived iNKT cells. **(C)** The integration of HLA-C and HLA-E (left) and the expression of *HLA-C* and *HLA-E* (right) in the WT, B2M-KO, and HLA-C or HLA-E re-expressed iPSC-derived iNKT cells. **(D)** The killing assay performed with HLA-edited iPSC-derived iNKT cells and NK cells from four individual donors. Data from two different HLA-C- and HLA-E- edited clones and the positive control K562, an HLA Class I negative leukemia cell line, and/or the control PBMC, are shown in the figure, *p < 0.05. The data are representative of two independent experiments **(B–D)**.

To evaluate whether re-expression of a single inhibitory HLA molecule is sufficient to protect iPSC-derived iNKT cells from NK cell-mediated cytotoxicity, we generated HLA-class I-deficient iPSCs by CRISPR-Cas9-mediated knockout of the *B2M* gene, followed by selective knock-in of HLA-C or HLA-E (Figure 1A). These genetically modified iPSC lines, including B2M-KO, HLA-C-re-expressed, and HLA-E-re-expressed, were differentiated into iNKT cells under on-feeder conditions and confirmed by flow cytometry (Figure 1B). Genomic integration of *HLA-C* or *HLA-E* was confirmed by PCR analysis (Figure 1C, left), while surface expression was validated by flow cytometry and compared with wild-type (WT) controls (Figure 1C, right). We next examined the effect of single HLA-C or HLA-E recapitulation on enhancing the resistance of iPSC-derived iNKT cells to NK cell-mediated cytotoxicity using a 24-h co-culture assay with NK cells from four independent donors (Figure 1D). These findings indicate that recapitulation of either HLA-C or HLA-E provides iPSC-derived iNKT cells with only limited protection from NK cell-mediated lysis compared to B2M-KO iPSC-derived iNKT cells.

### Dual HLA molecule recapitulation protects iPSC-derived iNKT cells from NK-mediated cytotoxicity

**FIGURE 2:**
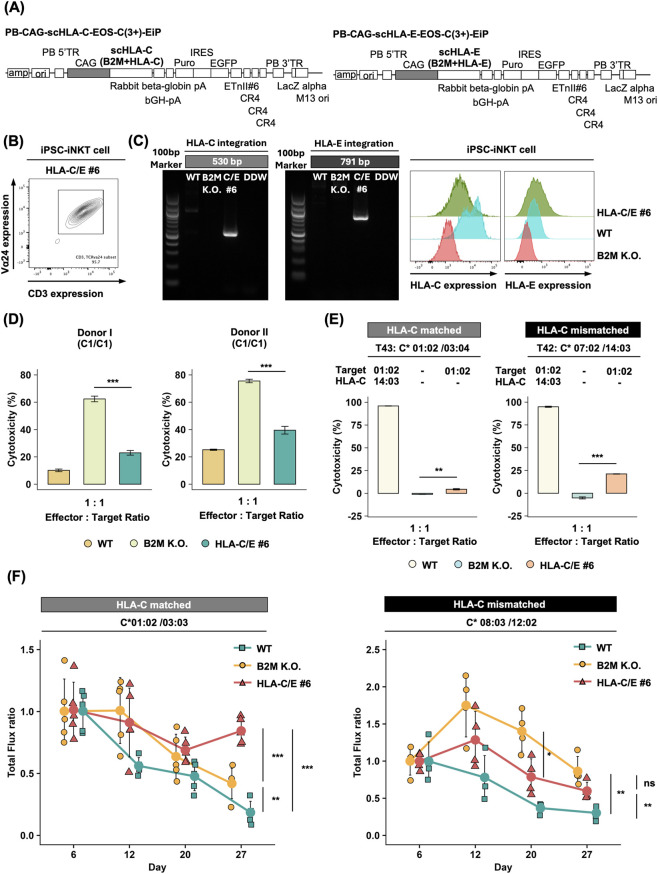
Dual HLA-C/E recapitulation provides antigenicity to T cells and increases *in vivo* residence time only in HLA-C allele-matched T cells. **(A)** Schematic of the piggyBac transposase and HLA-C or HLA-E transgene plasmids. **(B)** The expression of iPSC-derived iNKT cells markers CD3 and Vα24 on the HLA-C/E dual-expressing iPSC-derived iNKT cells clone #6. **(C)** The integration (left) and the expression of *HLA-C* and HLA-E (right) by iPSC-derived iNKT cells: HLA-C/E dual-expressing clone #6, WT, and B2M-KO. **(D)** A killing assay performed with WT, B2M-KO, and HLA-C/E dual-expressing iPSC-derived iNKT cells and NK cells from two individual C1/C1 allotype donors, ***p < 0.001. **(E)** A killing assay performed with WT, B2M-KO, and HLA-C/E dual-expressing iPSC-derived iNKT and T cells from two individual C1/C1 allotype donors carrying matched HLA-C (C*01:02/03:04, left) and mismatched HLA-C (C*07:02/14:03, right), **p < 0.01, ***p < 0.001. **(F)** The weekly change in luminescence of WT, B2M-KO, or HLA-C/E dual-expressing iPSC-derived iNKT cells that were co-transplanted with matched (left) or mismatched (right) HLA-C allele T cells from two donors, **p < 0.01, ***p < 0.001. The data are representative of two independent experiments **(B–F)**.

Since the recapitulation of a single HLA-class I provides only limited protection for B2M-KO iPSC-derived iNKT cells against NK cell missing-self cytotoxicity, we hypothesized that the dual recapitulation of HLA-C and HLA-E would provide an integrated inhibitory profile that dampens NK-cell activation. To validate this hypothesis, we first generated B2M-KO iPSCs engineered to simultaneously express HLA-C and HLA-E using the piggyBac transposon system (Figure 2A). WT and HLA-engineered iPSC lines, including B2M-KO and HLA-C/E dual-integrated variants, were then differentiated into iPSC-derived iNKT cells (shown for HLA-C/E in Figure 2B). Integration of *HLA-C* and *HLA-E* was confirmed by genomic PCR (Figure 2C, left). Recapitulation of HLA-C and HLA-E cell surface expression in differentiated B2M-KO iPSC-derived iNKT cells was confirmed by flow cytometry (Figure 2C, right).

Next, we evaluated NK-cell-mediated cytotoxicity by co-culturing WT, B2M-KO, and HLA-C/E iPSC-derived iNKT cells individually with NK cells from two independent C1/C1 allotype donors in a 24-h killing assay. As the engineered iPSC-derived iNKT cells re-express HLA-C*01:02, a member of the HLA-C1 ligand group, NK cells from C1/C1 donors were selected to provide a defined KIR-HLA-C interaction context, as HLA-C1 ligands are recognized by inhibitory KIRs such as KIR2DL2/3, allowing controlled assessment of HLA-C-mediated NK cell inhibition. The results revealed that simultaneous restoration of HLA-C and HLA-E substantially increased resistance to NK cell-mediated lysis in B2M-KO iPSC-derived iNKT cells (Figure 2D).

### HLA-C compatibility determines T-cell-mediated cytotoxic responses to HLA-C/E dual-expressing iPSC-derived iNKT cells

HLA-C compatibility between donor and recipient is reported to have a major effect on CD8^+^ T cell alloreactivity, as mismatched HLA-C alleles alter peptide presentation at the cell surface that triggers alloreactive CD8^+^ T cell activation (Petersdorf et al., 2014; Bettens et al., 2016).

Given the risk that HLA-C incompatibility may lead to the activation of recipient CD8^+^ T cells and graft failure, we next investigated whether HLA-C allele matching modulates T-cell-mediated cytotoxicity directed toward HLA-C/E iPSC-derived iNKT cells. To minimize background alloreactive responses and precisely determine the impact of HLA-C compatibility on recipient T cell activation, we generated HLA-reactive T cells by isolating CD8^+^ T cells from two donors carrying HLA-C alleles that either matched (C*01:02/03:04) or mismatched (C*07:02/14:03) the allele present on the WT iPSC-iNKT cells. Next, we compared the cytotoxicity of matched and mismatched target-specific T cells against HLA-C/E dual-expressing iPSC-derived iNKT cells in a 24-h killing assay. The dual recapitulation of HLA-C and HLA-E increased the susceptibility of iPSC-derived iNKT cells to T-cell-mediated killing, compared with B2M-KO cells (Figure 2E). However, this increase was smaller when the re-expressed HLA-C allele was compatible than when it was incompatible. These findings demonstrate that HLA-C compatibility critically affects CD8^+^ T cell-mediated alloresponses and suggest the importance of considering HLA-C allele variations when developing universally hypoimmunogenic cell therapies.

### Compatible HLA-C extends the *in vivo* residence time of HLA-C/E dual-expressing iPSC-iNKT cells

To further evaluate whether HLA-C compatibility affects the graft residence time of HLA-C/E dual-expressing iPSC-derived iNKT cells, we examined the survival of HLA-edited iPSC-derived iNKT cells *in vivo* in the presence of T cells. iPSCs were engineered to express Akaluc for Akaluc-based bioluminescence imaging, and the corresponding HLA modifications were introduced into these iPSCs. The generated Akaluc-expressing iPSC-derived iNKT cells, including WT, B2M-KO, and HLA-C/E dual-expressing cells, were transplanted intravenously into immunodeficient human IL-7/IL-15 expressing NSG mice, followed by administration of target-specific T cells carrying either a matched HLA-C allele (C*01:02/03:03) or a mismatched allele (C*08:03/12:02). Residence time was monitored using an IVIS imaging system until 27 days post-transplantation. Consistent with our *in vitro* findings, dual HLA-C and HLA-E recapitulation significantly extended graft survival of iPSC-derived iNKT cells under matched HLA-C conditions, as assessed by bioluminescence imaging (Figure 2F). At day 27 post-transplantation, HLA-C/E dual-expressing iPSC-derived iNKT cells exhibited significantly greater persistence compared with both WT iPSC-derived iNKT cells (p = 5.1 × 10^−6^) and B2M-KO iPSC-derived iNKT cells (p = 3.6 × 10^−4^), while WT iPSC-derived iNKT cells also showed reduced persistence relative to B2M-KO iPSC-derived iNKT cells (p = 8.9 × 10^−3^). In contrast, under mismatched HLA-C conditions, the protective effect of dual HLA-C/E recapitulation was diminished. Although B2M-KO iPSC-derived iNKT cells showed significantly greater persistence than HLA-C/E dual-expressing cells at day 20 (p = 1.5 × 10^−2^), no significant difference was observed between these groups at day 27 (p = 6.2 × 10^−2^). Furthermore, both WT iPSC-derived iNKT cells and B2M-KO iPSC-derived iNKT cells displayed significantly greater persistence than HLA-C/E dual-expressing cells at day 27 (p = 8.1 × 10^−3^ and p = 2.3 × 10^−3^, respectively). These results indicate that dual HLA-C and HLA-E restoration enhances graft survival of B2M-KO iPSC-derived iNKT cells only when the iPSC-derived iNKT cells and recipient share a compatible HLA-C allele, revealing the importance of considering HLA-C allele matching to optimize engraftment and therapeutic efficacy.

## Discussion

Universal iPSC-derived immune cells offer remarkable therapeutic potential but remain limited by allogeneic rejection mediated by host T and NK cells due to HLA incompatibility. Here, we demonstrate that selective restoration of HLA class I genes enhances the immune compatibility of HLA class I-deficient iPSC-derived iNKT cells. While single-molecule restoration of HLA-C or HLA-E offered limited protection from missing-self NK activation, dual HLA-C/E recapitulation generated a combined inhibitory signal that effectively suppressed NK cell-mediated cytotoxicity. In light of the pivotal role of HLA-C compatibility in modulating recipient CD8^+^ T cell alloreactivity, we further found that HLA-C/E dual-expressing iPSC-derived iNKT cells avoided T cell-mediated killing *in vitro* only under HLA-C-matched conditions. These findings extended to the *in vivo* system, where dual HLA-C/E recapitulation also prolonged graft survival under HLA-C-matched conditions. Together, these findings highlight a fundamental trade-off in HLA engineering for allogeneic cell therapy. While restoration of HLA-C and HLA-E reduces susceptibility to NK cell-mediated missing-self responses, re-expression of HLA-C can simultaneously reintroduce CD8^+^ T cell alloreactivity. Accordingly, we propose that dual HLA-C/E recapitulation provides important insight into the development of hypoimmunogenic iPSC-derived immune cell therapies with broad clinical potential.

Recapitulation of selective HLA class I molecules shows promise in mitigating NK cell-mediated cytotoxicity triggered by engineered HLA class I-deficient hypoimmunogenic stem cell lines. Previous studies have demonstrated that selective re-expression or retention of inhibitory HLA class I molecules can attenuate NK cell-mediated lysis caused by missing-self recognition. Specifically, enforced expression of HLA-E (Gornalusse et al., 2017), retention of HLA-C following disruption of HLA-A and -B (Xu et al., 2019), or introduction of the non-classical HLA-G in broadly HLA-deficient cells (Han et al., 2019) has been shown to suppress NK cell-mediated cytotoxicity. Beyond selective HLA restoration, complementary immune-evasion strategies, including CD47 upregulation to suppress macrophage-mediated phagocytosis and immune checkpoint engineering to modulate T cell activation, have also been explored to mitigate allogeneic immune rejection (Han et al., 2019). While these approaches show promise, each carries distinct safety and regulatory considerations. In parallel, we evaluated whether restoration of a single HLA class I molecule was sufficient to protect B2M-KO iPSC-derived iNKT cells from NK cell-mediated cytotoxicity. Our results revealed a limitation of single HLA class I restoration, suggesting that iPSC-derived iNKT cells may require more comprehensive or combinatorial HLA engineering approaches to effectively evade NK cell immune surveillance.

Overexpression of immunomodulatory factors or retention of dual HLA-class I molecules in engineered hypoimmunogenic cells dampens the NK-cell-mediated missing-self response (Han et al., 2019; Furukawa et al., 2023). Considering that HLA-C interacts with KIR family receptors and that HLA-E engages the CD94/NKG2A inhibitory complex to restrain NK-cell activation, we investigated whether co-expression of both molecules in B2M-KO iPSC-iNKT cells could provide a broader inhibitory signal. Our findings demonstrate that dual HLA-C/E expression provides an integrated inhibitory signal to NK cells and further imply that optimal HLA-editing strategies should consider the immunogenic properties of iPSC-derived immune cells.

Dual HLA-C/E recapitulation enabled iPSC-iNKT cells to evade NK-cell-mediated cytotoxicity, consistent with previous studies showing that HLA-C retention improves the immunocompetence of engineered iPSC lines, whereas HLA-C mismatch can trigger NK cell-mediated allogeneic responses (Ichise et al., 2017; Kitano et al., 2022). Beyond the NK-cell-mediated immune reaction, our findings revealed a distinct T-cell-mediated barrier, as HLA-C compatibility remains a critical determinant of graft survival. In HLA-C-matched recipients, dual HLA-C/E expression protected iPSC-iNKT cells from CD8^+^ T-cell-mediated cytotoxicity, and our *in vivo* results demonstrated correspondingly improved graft survival under HLA-C-matched conditions. Thus, while our approach significantly improves immune compatibility, the observed HLA-C-compatibility dependency remains a significant practical limitation for achieving durable engraftment and extended residence time.

To address this limitation and evaluate the clinical applicability of our approach, we further estimated, based on the information about allele frequency from the HLA laboratory (https://hla.or.jp/med/frequency_search/en/allele/), that only 10 iPSC lines with matched HLA-C alleles (*01:02, *03:03, *07:02, *03:04, *12:02, *08:01, *14:02, *14:03, *04:01, *15:02) would provide >95% coverage of the Japanese population. This suggests that a limited bank of iPSC lines carrying selected HLA-C alleles could achieve broad population coverage and supports the feasibility of a bank-based matching strategy. Practical implementation of an HLA-matched iPSC bank will require careful consideration of population diversity, regulatory frameworks, and long-term safety. Although our analysis supports feasibility in the Japanese population, extending this strategy globally will require population-specific allele frequency analyses. In addition, regulatory oversight will be essential to ensure genomic stability, transgene safety, and consistent immunogenicity profiles across iPSC lines. Nevertheless, advances in iPSC banking, genome editing, and standardized cell manufacturing infrastructure make such an approach increasingly feasible.

Several limitations of this study should be acknowledged. First, NK and T cell responses are inherently variable across donors, reflecting differences in KIR repertoires, HLA alleles, and T cell receptor diversity, which may influence the magnitude of the cytotoxic responses observed. Second, although the humanized NSG mouse model enables assessment of human T cell-mediated rejection, it does not fully recapitulate the complexity of human immune interactions, particularly NK cell education, tissue residency, and long-term immune dynamics. Third, while our study demonstrates HLA-C-dependent CD8^+^ T cell-mediated cytotoxicity, we did not directly assess peptide-specific T cell responses, which may further shape alloreactivity in clinical settings. Future studies incorporating more physiologically representative immune models and antigen-specific T cell analyses will be important to refine these findings.

Moreover, this study was conducted using a single established iPSC-derived iNKT cell line, and immune-compatibility assays were performed with a limited number of donor combinations. Although this proof-of-concept approach allowed controlled evaluation of HLA-C/E engineering, future studies should validate these findings using iPSC-derived iNKT cells generated from multiple independent iPSC donors and tested across a broader range of recipient immune donors. Donor CMV serostatus, which can shape NK- and T-cell repertoires, was not controlled for in this study and may have contributed to inter-donor variability in immune responses. Future studies incorporating CMV-stratified donor cohorts will be important to further refine immune-compatibility assessments.

In conclusion, this study identified T cell-mediated alloreactivity as a major barrier to allogeneic iPSC-iNKT engraftment and demonstrated that dual HLA-C/E restoration enhances immune compatibility when matched to recipient HLA-C alleles. This strategy enables the generation of HLA-C/E dual-expressing iPSC-iNKT cells from a limited master cell bank, providing a scalable approach for universal, off-the-shelf immunotherapies with improved graft survival. From a translational perspective, our findings have direct implications for the development of off-the-shelf CAR-iNKT and other engineered immune cell therapies. iNKT cells are particularly attractive for allogeneic applications due to their innate-like properties and reduced risk of graft-versus-host disease. Incorporating dual HLA-C/E restoration into CAR-iNKT platforms may enhance persistence and efficacy *in vivo* while maintaining safety. Importantly, the ability to derive such cells from a limited number of master iPSC lines supports scalable manufacturing and standardized quality control, which are essential for clinical translation.

## Data Availability

The original contributions presented in the study are included in the article/Supplementary Material, further inquiries can be directed to the corresponding author.
