# Supplementary material for: Extending the residence period of allogeneic iPSC-derived invariant natural killer T cells in humanized mice by editing HLA class I expression
Source: Front Cell Dev Biol. 2026 Jul 6;14:1869698. doi: 10.3389/fcell.2026.1869698 (PMC13381827; doi:10.3389/fcell.2026.1869698)
Supplement: Supplementary file 1 [file DataSheet1.pdf]

Extending the residence period of allogeneic iPSC-derived invariant natural killer T cells in humanized mice by editing HLA class I expression.

Yun-Hsuan Chang[1]*, Takahiro Aoki[1,3]*, Momoko Okoshi[1], Munechika Yamaguchi[1], Hiroko Okura[1], Satoko Sasaki[1], Yoshie Sasako[1], Sachiko Kira[1], Nayuta Yakushiji-Kaminatsui[1], Masashi Matsuda[1], Manabu Nakayama[2], Shinichiro Motohashi[3], Haruhiko Koseki[1]**

[1] Laboratory for Developmental Genetics, RIKEN Center for Integrative Medical Sciences, Kanagawa, JP

[2] Laboratory of Medical Omics Research, Department of Frontier Research and Development, Kazusa DNA Research Institute, Chiba, JP

[3] Department of Medical Immunology, Graduate School of Medicine, Chiba University, Chiba, JP

* Authors contributed equally

**** Correspondence:**

Haruhiko Koseki, MD, PhD

Laboratory for Developmental Genetics, RIKEN Center for Integrative Medical Science, Kanagawa, Japan.

1-7-29 Suehiro-cho, Tsurumi-ku, Yokohama, Kanagawa, 230-0045, Japan.

Tel.: +81-45-503-7059

E-mail: haruhiko.koseki@riken.jp

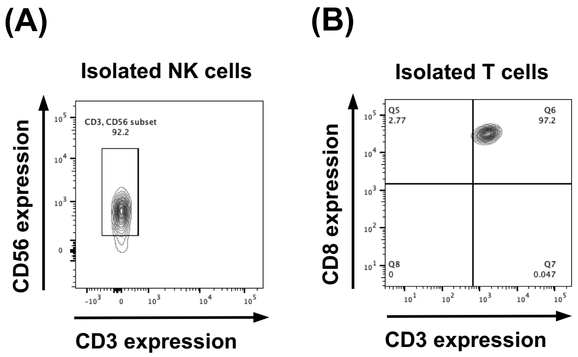

Supplementary Figure 1. (A) The expression of NK cell markers CD3 and CD56 on the isolated NK cells and non-NK cells. **(B)** The expression of $CD8^+$ T cell markers CD3 and CD8 on the iPSC-derived iNKT cells-reactive $CD8^+$ T cells.
